# Dr. John D. Mitchell

**Published:** 1915-08

**Authors:** 


**Dr. John D. Mitchell,** (not listed in Polk or State Directory), died at his home in Hornell, June 27, aged 62, suddenly but after long illness affecting the heart. He was a brother of Dr. S. Mitchell of Hornell and father of Dr. Wm. Mitchell of Weiser, Idaho.

